# Impacts of representing sea-level rise uncertainty on future flood risks: An example from San Francisco Bay

**DOI:** 10.1371/journal.pone.0174666

**Published:** 2017-03-28

**Authors:** Kelsey L. Ruckert, Perry C. Oddo, Klaus Keller

**Affiliations:** 1 Earth and Environmental Systems Institute, The Pennsylvania State University, University Park, Pennsylvania, United States of America; 2 Department of Geosciences, The Pennsylvania State University, University Park, Pennsylvania, United States of America; 3 Department of Engineering and Public Policy, Carnegie Mellon University, Pittsburgh, Pennsylvania, United States of America; Bristol University/Remote Sensing Solutions Inc., UNITED STATES

## Abstract

Rising sea levels increase the probability of future coastal flooding. Many decision-makers use risk analyses to inform the design of sea-level rise (SLR) adaptation strategies. These analyses are often silent on potentially relevant uncertainties. For example, some previous risk analyses use the expected, best, or large quantile (i.e., 90%) estimate of future SLR. Here, we use a case study to quantify and illustrate how neglecting SLR uncertainties can bias risk projections. Specifically, we focus on the future 100-yr (1% annual exceedance probability) coastal flood height (storm surge including SLR) in the year 2100 in the San Francisco Bay area. We find that accounting for uncertainty in future SLR increases the return level (the height associated with a probability of occurrence) by half a meter from roughly 2.2 to 2.7 m, compared to using the mean sea-level projection. Accounting for this uncertainty also changes the shape of the relationship between the return period (the inverse probability that an event of interest will occur) and the return level. For instance, incorporating uncertainties shortens the return period associated with the 2.2 m return level from a 100-yr to roughly a 7-yr return period (∼15% probability). Additionally, accounting for this uncertainty doubles the area at risk of flooding (the area to be flooded under a certain height; e.g., the 100-yr flood height) in San Francisco. These results indicate that the method of accounting for future SLR can have considerable impacts on the design of flood risk management strategies.

## Introduction

The warming climate is causing sea levels to rise around the globe [1–4]. As sea levels rise, settlements and ecosystems in low-lying coastal areas become more vulnerable to flooding. Currently, about 10% of the world’s population resides within 10 meters of present-day sea level [5] and about 40 million people are exposed to a 100-yr (also known as the 1-in-100 year or 1% annual exceedance probability) storm surge [6]. Rising sea levels drive hazards for transportation and energy facilities, tourism, agriculture, and human lives [7]. These hazards motivate the design and implementation of flood risk management strategies around the world [8, 9]. The strategies and infrastructures that manage flood risks are often designed for very low annual flooding probabilities, generally spanning the range from 1-in-50 to 1-in-10,000 years [10, 11]. Designing these strategies requires information about both storm surge and sea levels [10, 12].

Future projections of sea-level rise (SLR) are deeply uncertain [12–14]. Deep uncertainty in this context means that, “the suite of all possible future events as well as their associated probability distributions are … uncertain,” [15]. Sea-level projections are deeply uncertain because of the complex mechanisms controlling changes in sea level (i.e., thermal expansion of water, inputs from glaciers and ice sheets, changes in land water storage, glacial isostatic adjustment, vertical land motion from tectonics, and coastal erosion) [1, 16].

Many studies evaluate potential future flood risks (e.g., [7, 8, 17–23]). These studies provide valuable insights, but are often silent on the effects of known uncertainties surrounding SLR projections. For example, these studies often use the mean, best, or large quantile SLR estimate [7, 17–24]. More recent studies [25–27] have incorporated SLR distributions. Here, we expand on these studies by analyzing the question of how accounting for (an estimate of) the full distribution of SLR can impact flood risks as well as the design of flood risk management strategies. First, we explicitly show that neglecting uncertainty about SLR projections can result in considerable underestimation of flood risks. Second, we demonstrate and quantify how accounting for the full distribution changes the shape of the survival function. The survival function, also referred to as the exceedance probability, is one minus the cumulative frequency [28]. Last, but not least, we put these results into a coastal risk management perspective. We demonstrate these effects for the San Francisco Bay (SFB) area using a previous study design [7, 17].

## Methods

### Choice of case study

**Fig 1:**
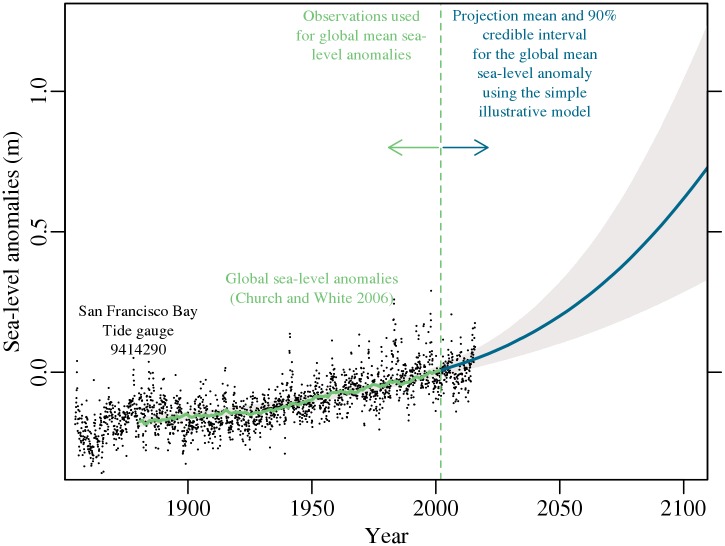
Comparison of historical sea-level anomalies in San Francisco Bay (SFB) to global mean sea-level anomalies and projections. The black dots represent the monthly mean sea level at the SFB tide gauge [31]. Note that the SFB tide gauge observations are not used in the global mean sea level modeling process. The green line represents synthesized global mean sea-level anomalies relative to the mean sea level in the year 2000 [32], where the gray envelope is the 90% credible interval and the blue line is the projected mean fitted to those anomalies.

We demonstrate the effect of accounting for uncertainty in the SFB area of California. We choose California as an area of interest, because California has more than 2,000 miles of coastline with roughly 32 million people living in coastal watershed counties [7, 17, 29, 30]. In particular, the SFB area is a useful case study for three reasons. First, we can compare our results to an existing analysis of the California coastline [7, 17]. More importantly, the SFB area has a long and complete hourly tide record (>100 years with no missing data) and available, relatively high-resolution digital elevation models. Lastly, SLR in the SFB area has changed about as much as global mean sea level over the past 100 years (Fig 1).

### Projecting sea-level rise

To demonstrate the effect of representing SLR uncertainty, we approximate the methods found in an existing analysis of the California coastline [7]. This previous analysis [7] uses global mean SLR as a proxy for local SLR. As shown in Fig 1, this assumption seems reasonable for the SFB area. Following this previous study design [7], we hindcast and project SLR using a global mean sea-level model [1]. However, we do not adapt SLR estimates to account for future changes in water stored behind dams and in reservoirs (see S8–S11 Figs which account for this change in land water storage). The global mean sea-level model [1] predicts global mean SLR on an annual time step using estimated global mean sea-level anomalies [32] and global mean surface air temperatures *T* (°C). Historical temperatures are based on observations of merged sea surface and land-near surface temperature anomalies [33, 34]. Projections are based on the CNRM-CM5 simulation of the RCP8.5 scenario from the CMIP5 multi-model ensemble; http://cmip-pcmdi.llnl.gov/cmip5/. In the model, the rate of global mean SLR *H* at time *t*, *t* from 1880 to the year 2300, is approximated as the sensitivity of sea level to global mean temperature *α* times the difference between temperature at time *t* and the temperature when the sea-level anomaly equals zero *T*_0_,

$$\begin{array}{c} \frac{\delta H_{t}}{\delta t}=\alpha \left(T_{t}-T_{0}\right). \end{array}$$ (1)

Following a Bayesian approach (as described in detail in [35]), we fit the model to global sea-level anomalies with respect to the average sea level in 2000 [32] using a Markov chain Monte Carlo method accounting for interdependent (autocorrelated) residuals and time-varying (heteroskedastic) observation error [36–38]. Using this method, we approximate the noisy observations *y*_*t*_ as the sum of the semi-empirical model simulations *H*_*t*_, residuals *R*_*t*_ (i.e., approximating the effects of unresolved internal variability and model error), and observation errors *ϵ*_*t*_,

$$\begin{array}{c} y_{t}=H_{t}+R_{t}+\epsilon_{t}, \end{array}$$ (2)

$$\begin{array}{c} R_{t}=\rho \times R_{t-1}+\delta_{t}, \end{array}$$ (3)

$$\begin{array}{c} R_{1}∼N\left(0,\frac{\sigma_{AR1}^{2}}{1-\rho^{2}}\right),\delta_{t}∼N\left(0,\sigma_{AR1}^{2}\right),\epsilon_{t}∼N\left(0,\sigma_{\epsilon ,t}^{2}\right), \end{array}$$ (4)

where *R*_*t*_ is a stationary first-order autoregressive process. It is characterized by an annual autoregression coefficient *ρ* and a white noise process *δ*_*t*_ with zero mean and constant variance $\sigma_{AR1}^{2}$. *ϵ*_*t*_ represents the observation errors (also known as measurement errors) with time-varying known variance $\sigma_{\epsilon ,t}^{2}$. We estimate the posterior density of the model parameters (*α*, *T*_0_, and *H*_0_) and the statistical parameters (*ρ* and *σ*_*AR*1_) with uniform prior distributions using the Markov chain Monte Carlo method and the Metropolis Hastings algorithm [37–40]. In this method, the likelihood function incorporates the variance of the autocorrelation process (accounting for the autocorrelated structure of the residuals) and the heteroskedastic observation error. We choose this implementation process because ignoring known observational properties (i.e., accounting for autocorrelated residuals and heteroskedastic errors) could lead to overconfident projections [35]. The observation errors are set as the reported measurement error values [32]. We use 2.5 × 10^7^ iterations. We assess convergence using visual inspection and the potential scale reduction factor [41], remove a 1% initial “burn-in” from the Markov chains, and thin the chains to subsets of 2 × 10^4^ for the analysis [38].

### Estimating current and future flood heights

**Fig 2:**
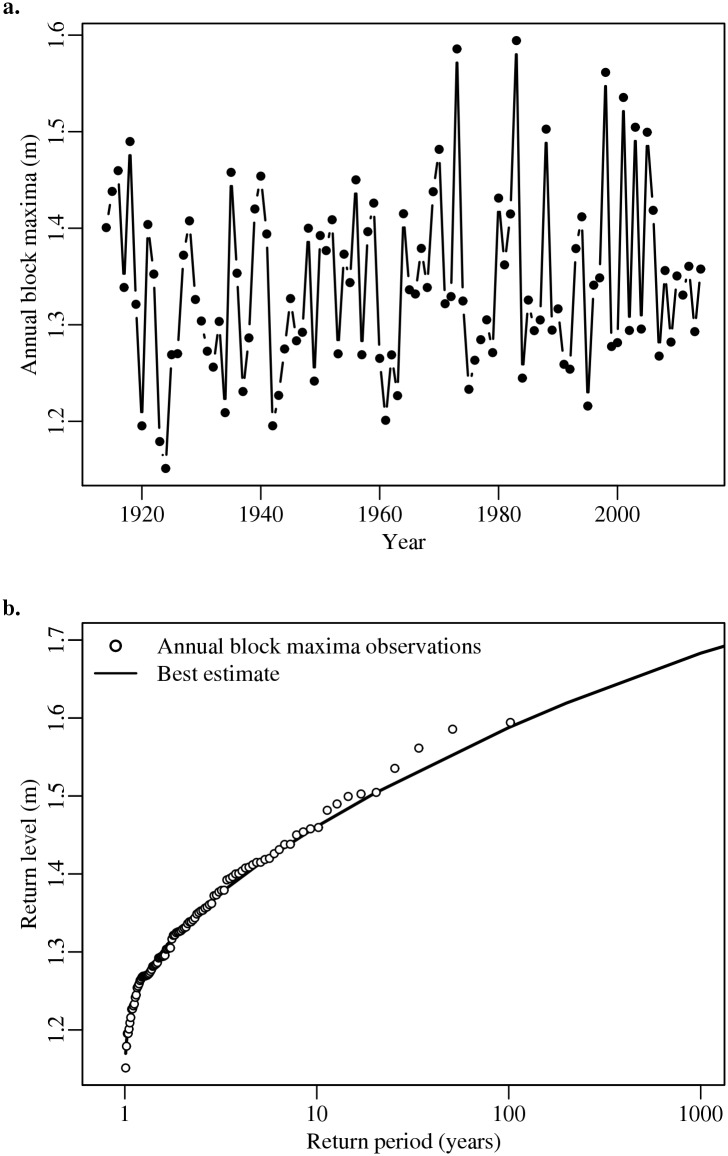
Storm surge analysis for San Francisco Bay area. Shown are (panel a) the maximum recorded sea-level anomaly in a year, the annual block maxima, and (panel b) the return levels for the San Francisco Bay tide gauge (in meters).

We approximate the baseline (current) 100-yr (i.e., the 1-in-100 year) storm surge for the SFB area using a Generalized Extreme Value (GEV) analysis [42] and analyzing hourly data from 1914 to 2014 for the San Francisco tide gauge [31] (the location of the tide gauge is shown in S1 Fig). We remove the longer-term signal (SLR) by subtracting the annual means from the record. The detrended values represent the impacts of day-to-day weather, tides, and seasons [43]. These detrended values are then grouped into non-overlapping annual observation periods. This process focuses the attention to the maximum observation in each year, the annual block maxima. We fit the annual block maxima to the GEV distribution for parameter estimation,

$$\begin{array}{c} F\left(x;\mu ,\sigma ,\xi \right)=\exp\{-{\left[1+\xi \left(\frac{x-\mu }{\sigma }\right)\right]}^{-1/\xi }\}, \end{array}$$ (5)

where the parameters *μ*, *σ*, and *ξ* control the location, scale, and shape of the distribution [42, 44–46]. Using the maximum likelihood estimate of the GEV parameters and a range of probabilities [44–46], we approximate the flood return levels (the height associated with a probability of occurrence) with a 95% confidence interval out to the 100,000-yr return period (the inverse probability that an event of interest will occur). We use the maximum likelihood estimate of the flood return levels as our baseline survival function (Fig 2; please see the discussion of this point in the Caveats section).

**Fig 3:**
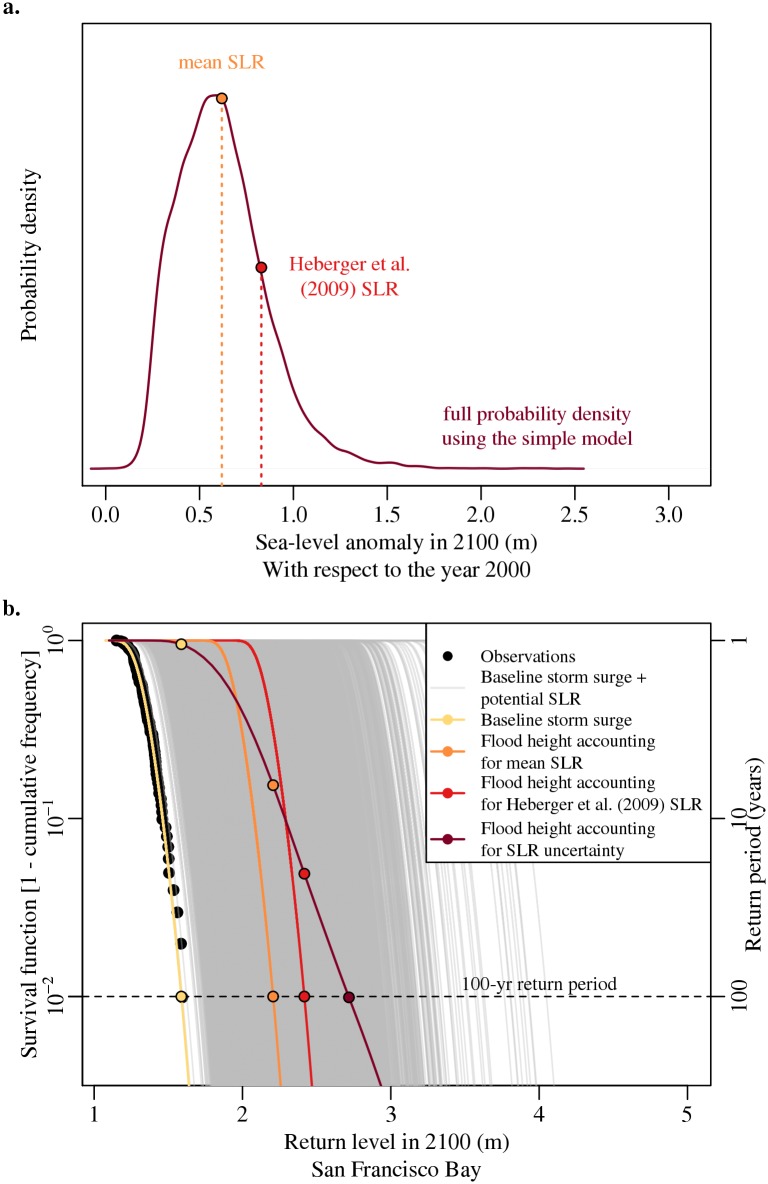
Estimated (panel a) probability density function of global mean sea-level rise in 2100 and (panel b) flood survival functions for San Francisco Bay. In panel a, the dark red line represents the sea-level distribution in the year 2100, whereas, the orange and red points display the mean and Heberger et al. [7] (not accounting for land storage changes) sea-level estimate. In panel b, the baseline survival function for San Francisco Bay (yellow) is shifted relative to increases in global mean sea level by the mean sea-level projection (orange), the Heberger et al. [7] estimate (0.8 m; red), and each individual sea-level projection from the distribution of Markov chain Monte Carlo samples (gray) for the year 2100. Accounting for sea-level uncertainty produces the survival function in dark red. The associated return period is displayed on the right axis. The distance between the points on the dashed line (100-yr return period) to the same color point on the dark red curve display the flood risk underestimation.

We approximate the potential future 100-yr flood height (storm surge including SLR) by accounting for the mean sea-level anomaly and SLR uncertainty in the year 2100. For the 100-yr flood height accounting for mean SLR, we add the mean sea-level anomaly (compared to the year 2000) to the baseline 100-yr storm surge. We use several steps to approximate the effects of future SLR uncertainty, extrapolate a flood survival function, and approximate the 100-yr flood height accounting for SLR uncertainty. First, we add each SLR estimate from the distribution for the year 2100 to the baseline survival function. This results in 2 × 10^4^ simulations of the future flood survival function (shown as the gray lines in Fig 3b). We then estimate the probability of a specific flood height occurring for each survival function simulation. If the probability falls in between two known values, we linearly interpolate the probability from the surrounding values. This results in 2 × 10^4^ probability estimates for a specific flood height. We then average the 2 × 10^4^ probability estimates for a specific flood height to approximate the actual probability for that return level. We replicate this process of estimating the return period for a range of flood heights from 1.1–3.3 m (see Caveats). This method produces a new flood survival function where the return level corresponding to a 1% probability of occurring in the year 2100 is taken to be the 100-yr flood height accounting for SLR uncertainty (the dark red curve and point in Fig 3b).

### Estimating flood inundation areas

We assess the area at risk of flooding (the area to be flooded under a certain height; e.g., the 100-yr flood height) with a geographic information system (ESRI ArcGIS Desktop) using 1/9-arc second (nominal resolution of ∼3 m) topobathy digital elevation models [47]. We use the mosaicked elevation data as inputs for a simple bathtub inundation model [22, 48]. The elevation data is adjusted to the 1983–2001 local tidal datum mean sea level [31]. Hence, any elevation below 0 m is considered to be current standing water (i.e., ocean, lakes, rivers, and reservoirs). We generate the 100-yr flood risk area by reclassifying the elevation values from 0 m to the estimated baseline storm surge or future flood height. The reclassified values are then extracted and converted from raster datasets to polygons. Using county subdivision shapefiles from the U.S. Census Bureau [49], we estimate the area at risk of flooding for San Francisco, Oakland, and Alameda. We replicate this process for each assumption about future flood height in the year 2100 (i.e., the 100-yr flood height accounting for mean SLR, the 100-yr flood height accounting for the Heberger et al. [7] sea-level estimate, and the 100-yr flood height accounting for SLR uncertainty). All of the data is set to the NAD 1983 California Zone 3 projection. We use the State plane coordinate system to minimize distance and area distortions [50].

## Results and discussion

### Future sea-level rise and flood height

Future changes in sea level increase the return level for the 100-yr flood height in the SFB area. Our application of the global mean sea-level model [1] suggests that sea level will rise by roughly 0.6 m by the year 2100, or between 0.3 and 1.1 m (mean and 90% credible interval) above the average sea level in 2000 (Fig 1). Currently, the baseline 100-yr storm surge in the SFB area is roughly 1.6 m (Fig 2). If sea level is assumed to rise by the mean estimate or the 90% estimate in 2100, then the 100-yr flood height would increase to a return level of roughly 2.2 m or 2.5 m, respectively (Fig 3 and S2 Fig). Accounting for sea-level uncertainty further increases this return level to roughly 2.7 m (Fig 3). Hence, projections that assume sea level will remain constant or will rise by the mean or 90% estimate, underestimate the return level associated with a 100-yr flood height by roughly 1.1 m, 0.5 m, and 0.2 m in the year 2100, respectively (Fig 3 and S2 Fig).

### Accounting for sea-level rise uncertainty can increase the probability of flood occurrence

Accounting for mean SLR underestimates the probability of flood occurrence in this specific case study. When we consider just the mean SLR, the 100-yr return period occurs at 2.2 m. If we account for the 90% SLR, the 100-yr return period occurs at 2.5 m (S2 Fig). Yet, when we account for sea-level uncertainty, the 100-yr return period increases to 2.7 m. By accounting for sea-level uncertainty, the return level of 2.2 m no longer has a 100-year return period. Instead, the probability of occurrence is higher as a 7-yr return period (∼15% probability) in the year 2100 (Fig 3). Additionally, the return level of 2.5 m moves from a 100-yr return period (for the 90% SLR assumption) to a roughly 33-yr return period (∼3% probability) in the year 2100 (S2 Fig).

Our results are broadly consistent with some previous findings (e.g., [26, 43, 51]). A simple calculation can demonstrate how we account for uncertainty and why accounting for uncertainty can increase the probability of flood occurrence for a return level (S3 Fig). For a very simple illustrative example, we describe how to estimate the probability associated with a flood height using three values from the SLR distribution. Note that for the analysis we use the entire distribution (2 × 10^4^ values) and repeat the process for multiple flood heights to produce a new survival function accounting for uncertainty. For the example, consider using three samples from the SLR distribution (i.e., the mean SLR—1 standard deviation, the mean SLR, and the mean SLR + 1 standard deviation). Adding those sea-level values to the baseline storm surge survival function results in three new survival functions with 100-yr flood heights of roughly 2.0, 2.2, and 2.5 m (S3b Fig). We can then estimate the expected probability of the 2.2 m flood height by averaging the probability of the 2.2 m flood height from each of the three new survival functions. The 2.2 m flood height has a probability of roughly 4 × 10^−6^ (mean SLR—1 standard deviation), 0.01 (mean SLR) and 0.4 (mean SLR + 1 standard deviation). The results from averaging the three samples produces a higher return period for a specific flood height when compared to estimates that neglect uncertainty (i.e., accounting for the mean estimate). In this specific case, the probability of flood occurrence at the 2.2 m flood height when considering uncertainty shortens the return period from a 100-yr to a roughly 7-yr return period (S3b Fig).

### The effects of accounting for sea-level rise are dependent on the sea-level rise distribution characteristics

These underestimated flood occurrences are due to the fact that accounting for the full distribution changes the shape of the survival function. We create a simple test to further investigate how incorporating the full distribution affects the survival function. For instance, we compare how the shape and characteristics of the SLR distribution impact the results by comparing our SLR distribution to a normal, log normal, and Pareto distribution (S4a Fig). Specifically, we approximate the data from a normal, log normal, and Pareto distribution using the mean and standard deviation estimates from our SLR distribution. As perhaps expected, the shape of the distribution impacts the resulting expected return level. The result shows that as the upper tail becomes fatter the return level increases and the shape of the survival function increases compared to the baseline survival function (S4b Fig). Hence, approximating the full distribution can cut off important tails which can lead to underestimating the return periods (Fig 3, S2 and S4 Figs).

### Accounting for uncertainties can impact adaptation strategies

**Fig 4:**
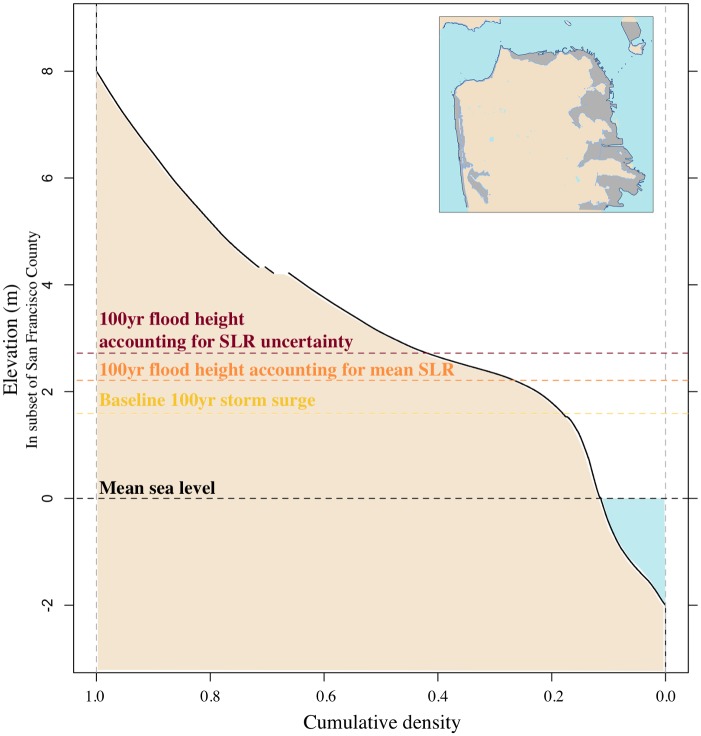
Hypsometric curve covering the elevations between -2 and 8 m in San Francisco County. The gray area within the inset plot displays the area analyzed in this curve. The area in blue is water, whereas the area in tan is land. The dashed lines represent the elevation associated with the mean sea level (black), the baseline 100-yr storm surge (yellow), and the future 100-yr flood height (orange and dark red). The black curve displays the cumulative density or percentage of the area analyzed at elevations between -2–8 m. For example, ∼42% of the area analyzed has an elevation of 2.7 m (100-yr flood height accounting for uncertain sea-level rise) or lower.

**Fig 5:**
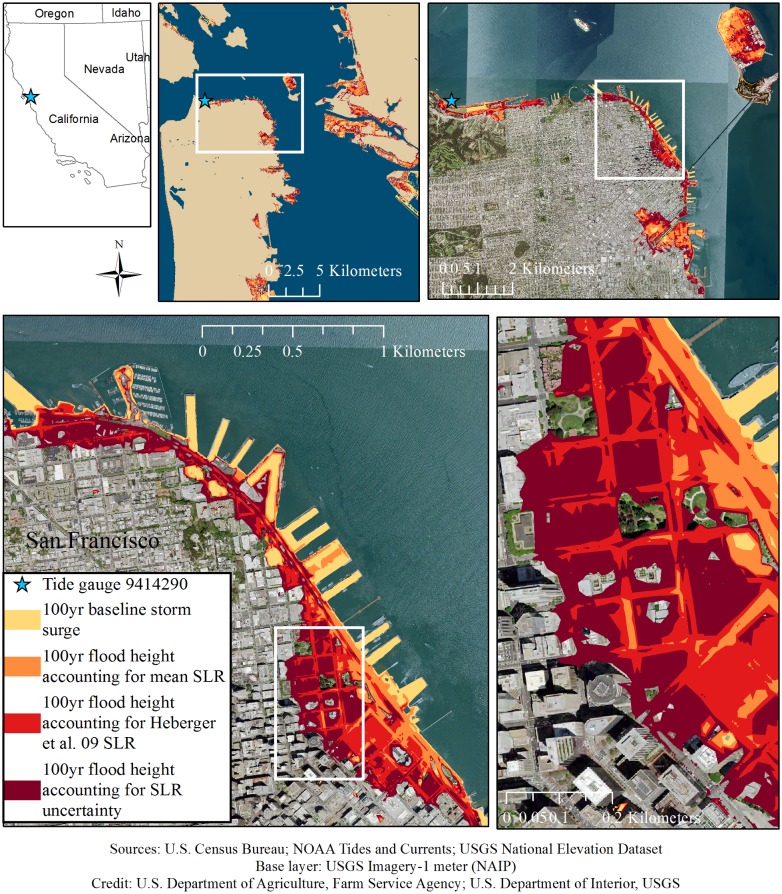
Sequential zoom in of the baseline and future 100-yr flood risk area in San Francisco. The maps display the baseline 100-yr flood risk area in yellow. In the year 2100, the potential 100-yr flood height includes accounting for the mean sea-level projection (orange), the Heberger et al. [7] (not accounting for land storage changes) sea-level projection (red), and accounting for sea-level rise uncertainty (dark red). The star is the location of the tide gauge.

Relatively small changes in the return level can impact adaptation strategies [7]. An increase in the return level expands the area at risk of flooding (the area analyzed is shown in S5 Fig). This expansion hinges on the change of elevation along the coast. For example, roughly 15% more of the area between -2–8 m elevation in San Francisco County has an elevation of up to 2.7 m (2.7 m refers to the 100-yr flood height accounting for uncertain SLR) versus up to 2.2 m (2.2 m refers to the 100-yr flood height accounting for mean SLR) (Fig 4). Additionally, the upper tail is—in this specific case—of primary importance, because an area is flooded if the water is above a certain limit (e.g., an opening in a house). The probability that the water level is above a certain threshold is the probability in the upper tail (see Fig 5). In this specific case, the area susceptible to flooding increases when we account for the tails in the SLR distribution. For example, in San Francisco County, the baseline 100-yr storm surge places roughly 1.6 km^2^ at risk of flooding (Fig 5 and Table 1). Accounting for SLR in 2100 expands the area at risk of flooding to roughly 3.9 km^2^ (using the mean SLR estimate) and roughly 8.0 km^2^ (accounting for uncertain SLR). In comparison, accounting for SLR uncertainty increases the mean SLR flood risk area by roughly a factor of 2 (Fig 5). In Oakland (S6 Fig) and Alameda (S7 Fig), accounting for SLR uncertainty increases the mean SLR flood risk area by roughly 65% and 75%, respectively. As the area at risk of flooding increases, more people and assets are exposed to the hazard. This increased exposure has implications for the design of flood risk management strategies.

**Table 1:** Comparison of flood risk area by the potential future 100-yr flood height accounting for different estimates of sea-level rise (SLR).

100-yr Flood risk area in county subdivisions (sq.km)				
County subdivisions	Baseline storm surge	Flood height accounting for mean SLR	Flood height accounting for Heberger et al. 2009 SLR	Flood height accounting for SLR uncertainty
*San* *Francisco*	1.6	3.9	5.5	8.0
*Oakland*	6.3	10.9	13.7	18.0
*Alameda*	1.9	6.4	8.4	11.2

### Caveats and future research needs

We use relatively simple models and statistical methods to make a simple point. This simplicity is chosen to provide a hopefully transparent analysis. Yet, this simplicity requires us to make several approximations that lead to caveats and future research needs. For example, we neglect uncertainty associated with the GEV parameters. Additionally, we use a simple interpolation method to estimate the inverse of the survival function using the surrounding values when accounting for SLR uncertainty. If the return level value does not exist, then a value of zero is returned as the probability of flood occurrence. Due to the tight shape, the return level values with probabilities of flood occurrence below the 100,000-yr return period are assigned to have a zero probability of flood occurrence. A simple test suggests the impact of this approximation is relatively small, but can potentially lead to conservative estimates (S3b and S3c Fig). It is also important to note that future mean SLR for the SFB area may differ from projections in this study. This is because our SLR model may potentially neglect effects such as the melting ice sheets (including marine ice sheet instability and cliff instability [16, 52]), glacial isostatic adjustment, vertical land motion from tectonics, or coastal erosion. Additionally, we do not address the possibility that future storm patterns could change in frequency or severity because of climate change. As a last example, this study assumes that no actions are taken to protect the coast. These caveats imply that this illustrative analysis is not to be used to assess actual coastal hazards.

## Conclusion

As sea levels rise, flood risks are projected to increase. Studies evaluating future flood risks are often silent on the impact of uncertainty in sea-level projections and instead consider the mean, best, or large quantile (i.e., 90%) estimate. We show how accounting for sea-level rise uncertainty can increase the area at risk of flooding and can increase the probability of flood occurrence. Using the San Francisco Bay area as an example, we demonstrate that these effects can be sizable. Specifically, we show how accounting for uncertainty increases the 100-yr return level by 0.5 m, shortens the return period from a 100-yr to a roughly 7-yr return period, changes the shape of the survival function, and roughly doubles the area at risk of flooding in San Francisco over using the mean sea-level rise estimate. Although, we use the San Francisco Bay area as an example the overall results are transferable to many regions and indicate that the method of accounting for sea-level rise can have considerable impacts on the design of flood risk management strategies.

## Supporting information

S1 Fig Proximity of tide gauge 9414290 to San Francisco, CA. Photograph of the tide gauge (bottom left corner) used in this study. Photograph by KL Ruckert on July 14, 2011.
(TIF)

S2 Fig Re-plot of Fig 3 including the 90% sea-level rise estimate and flood survival function for San Francisco Bay. As in Fig 3, panel a is the probability distribution of global mean sea-level rise in 2100 and panel b is the flood survival functions for San Francisco Bay.
(TIFF)

S3 Fig Illustrative example (panel a and b) of how accounting for uncertainty increases the probability of flood occurrence and (panel b and c) the impact of approximating probabilities of occurrence below the 100,000-yr return period. Panel a displays the probability density function of global mean sea-level rise in the year 2100 along with the mean ±1 standard deviation projections (green lines). In panel b, the baseline storm surge (light blue) is shifted by sea-level projections of 0.4 m (-1 standard deviation; light green), 0.6 m (mean; green), and 0.9 m (+1 standard deviation; dark green) to represent potential future flood height. The average of the three return periods at 2.2 m is represented as the black square. The pink star is produced when the return period below the 100,000-yr return period is approximated as zero and then averaged with the two other return periods at 2.2 m. Note that (panel b and c) the approximation method for the return period at 2.2 m produces the same return period value as the result from accounting for the return periods below 100,000 years and hence displays little to no flood risk over-or-underestimation.
(TIFF)

S4 Fig Test of the impact that sea-level distribution characteristics have on the survival function. Panel a displays the probability density function (pdf) of our global mean sea-level rise in the year 2100 (dark red) along with the normal (light blue), log normal (blue), and Pareto (dark blue) distribution approximations of global mean sea-level rise in the year 2100. In panel b, the survival function accounting for each sea-level rise approximation (light to dark blue) is shown for comparison to the baseline storm surge (yellow) and the survival function accounting for our estimated empirical sea-level rise pdf (dark red).
(TIFF)

S5 Fig Extent of county subdivisions within the area analyzed. Each county subdivision (outlined in brown) is located within the analysis extent. In the analyzed area, the 100-yr flood risk area is displayed in yellow (baseline), orange (flood height accounting for the mean sea-level projection), red (flood height accounting for the Heberger et al. [7] sea-level projection; not accounting for land storage changes), and dark red (flood height accounting for sea-level rise uncertainty). The star represents the location of the tide gauge.
(TIFF)

S6 Fig Sequential zoom in of baseline and future 100-yr flood risk areas in Oakland, CA. The maps display the baseline 100-yr flood risk area in yellow. In the year 2100, the potential 100-yr flood height includes the addition of sea-level projections. The future 100-yr flood risk area based on the mean sea-level projection is in orange, based on the Heberger et al. [7] (not accounting for land storage changes) sea-level projection in red, and based on accounting for sea-level rise uncertainty is in dark red. The location of the tide gauge is displayed as a star.
(TIFF)

S7 Fig Sequential zoom in of baseline and future 100-yr flood risk areas in Alameda, CA. The maps display the baseline 100-yr flood risk area in yellow. In the year 2100, the potential 100-yr flood height includes the addition of sea-level projections based on the mean sea-level projection (orange), the Heberger et al. [7] (not accounting for land storage changes) sea-level projection (red), and accounting for sea-level rise uncertainty in dark red. The location of the tide gauge is displayed as a star.
(TIFF)

S8 Fig Re-plot of Fig 3 where we adapt our sea-level rise estimates to account for future changes in water stored behind dams and in reservoirs. As in Fig 3, panel a is the probability distribution of global mean sea-level rise in 2100 and panel b is the flood survival functions for San Francisco Bay. However, in this figure we adapt our sea-level rise estimates in the year 2100 by adding roughly ∼0.55 m. This increase is roughly comparable to the increase used in Heberger et al. [7]. Ultimately, adapting sea-level estimates to this change does not change the main conclusions of this analysis.
(TIFF)

S9 Fig Re-plot of Fig 5 where we adapt our sea-level rise estimates to account for future changes in water stored behind dams and in reservoirs. As in Fig 5, this figure shows a sequential zoom in of the baseline and future 100-yr flood risk areas in San Francisco. However, in this figure we account for changes in land water storage in the year 2100 by adding roughly 0.55 m to the sea-level estimates [7].
(TIFF)

S10 Fig Re-plot of S6 Fig where we adapt our sea-level rise estimates to account for future changes in water stored behind dams and in reservoirs. The maps of Oakland, CA display the baseline 100-yr flood risk area in yellow. In the year 2100, the potential 100-yr flood height includes the addition of sea-level projections. The future 100-yr flood risk area based on the mean sea-level projection is in orange, based on the Heberger et al. [7] sea-level projection in red, and based on accounting for sea-level rise uncertainty is in dark red. The location of the tide gauge is displayed as a star. However, in this figure we account for changes in land water storage in the year 2100 by adding roughly 0.55 m to the sea-level estimates [7].
(TIFF)

S11 Fig Re-plot of S7 Fig where we adapt our sea-level rise estimates to account for future changes in water stored behind dams and in reservoirs. The maps of Alameda, CA display the baseline 100-yr flood risk area in yellow. In the year 2100, the potential 100-yr flood height includes the addition of sea-level projections based on the mean sea-level projection (orange), the Heberger et al. [7] sea-level projection (red), and accounting for sea-level rise uncertainty in dark red. The location of the tide gauge is displayed as a star. However, in this figure we account for changes in land water storage in the year 2100 by adding roughly 0.55 m to the sea-level estimates [7].
(TIFF)

S1 Table The CMIP modeling group whose model output we used in this study (archived at http://cmip-pcmdi.llnl.gov/cmip5/). This table was accessed and modified on 13 June 2016 from http://cmip-pcmdi.llnl.gov/cmip5/docs/CMIP5_modeling_groups.docx.
(PDF)
