# Supplementary figures and images for: Impacts of representing sea-level rise uncertainty on future flood risks: An example from San Francisco Bay
Source: PLoS One. 2017 Mar 28;12(3):e0174666. doi: 10.1371/journal.pone.0174666 (PMC5370151; doi:10.1371/journal.pone.0174666)

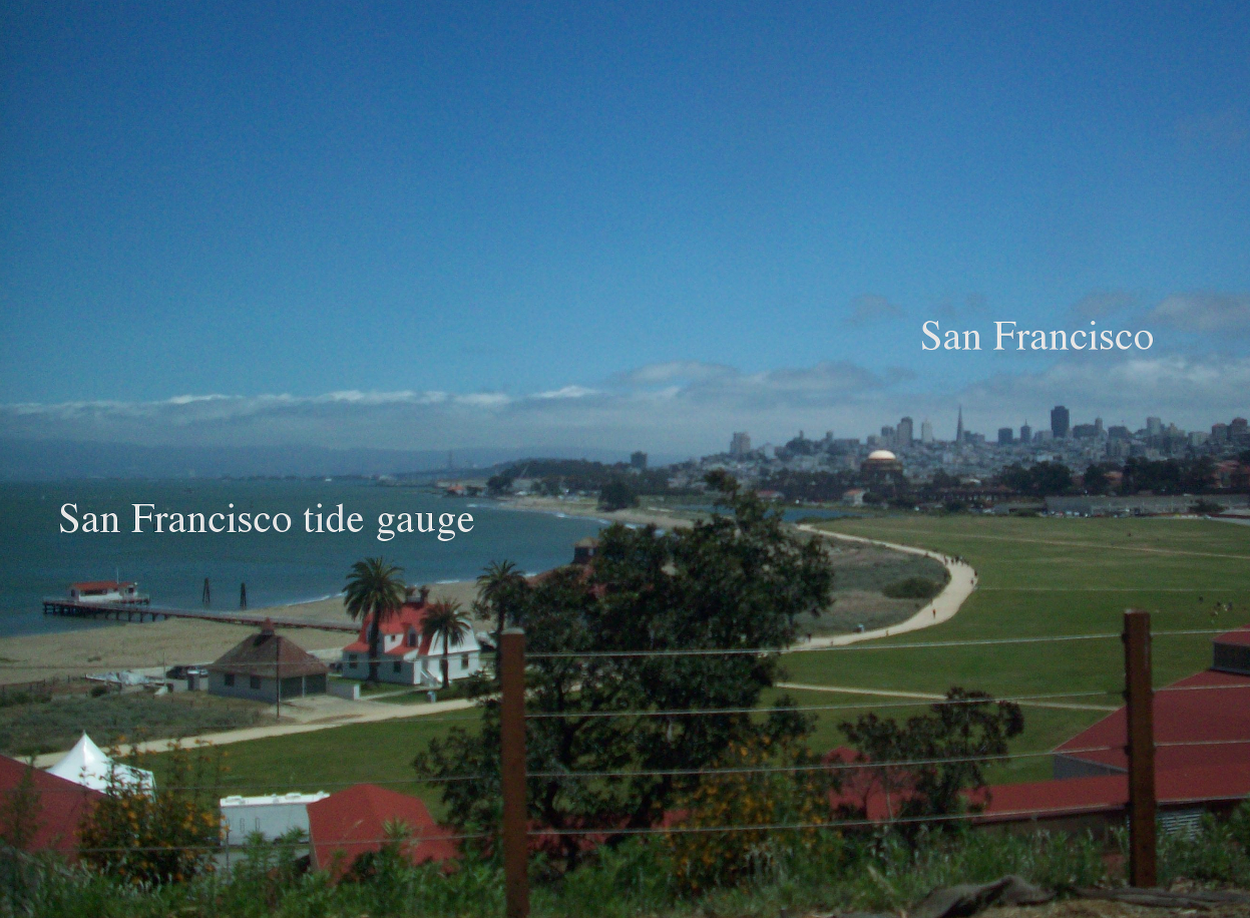

Supplement: S1 Fig — Photograph of the tide gauge (bottom left corner) used in this study. Photograph by KL Ruckert on July 14, 2011. (TIF) [file pone.0174666.s001.tif]

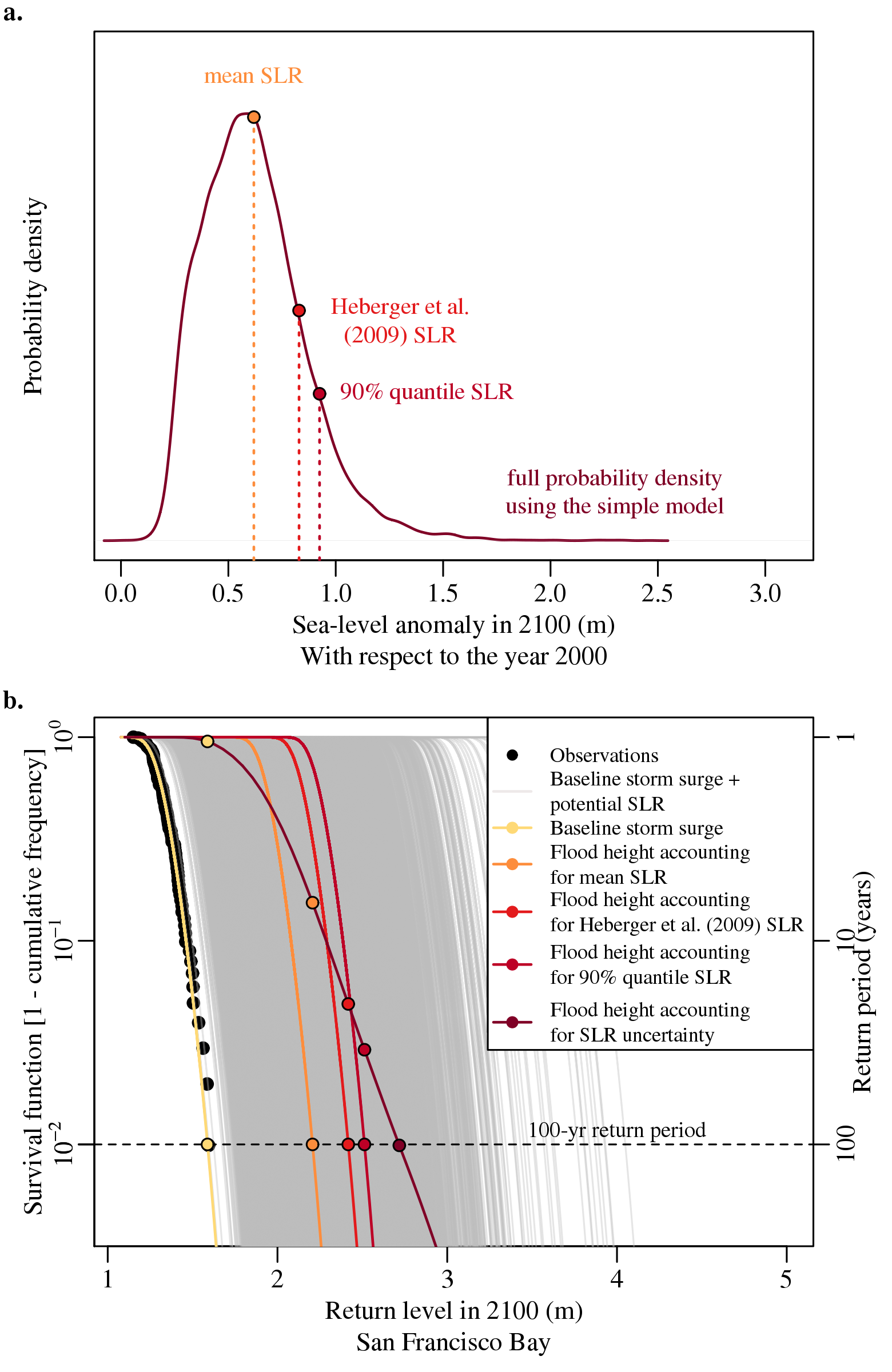

Supplement: S2 Fig — As in Fig 3, panel a is the probability distribution of global mean sea-level rise in 2100 and panel b is the flood survival functions for San Francisco Bay. (TIFF) [file pone.0174666.s002.tiff]

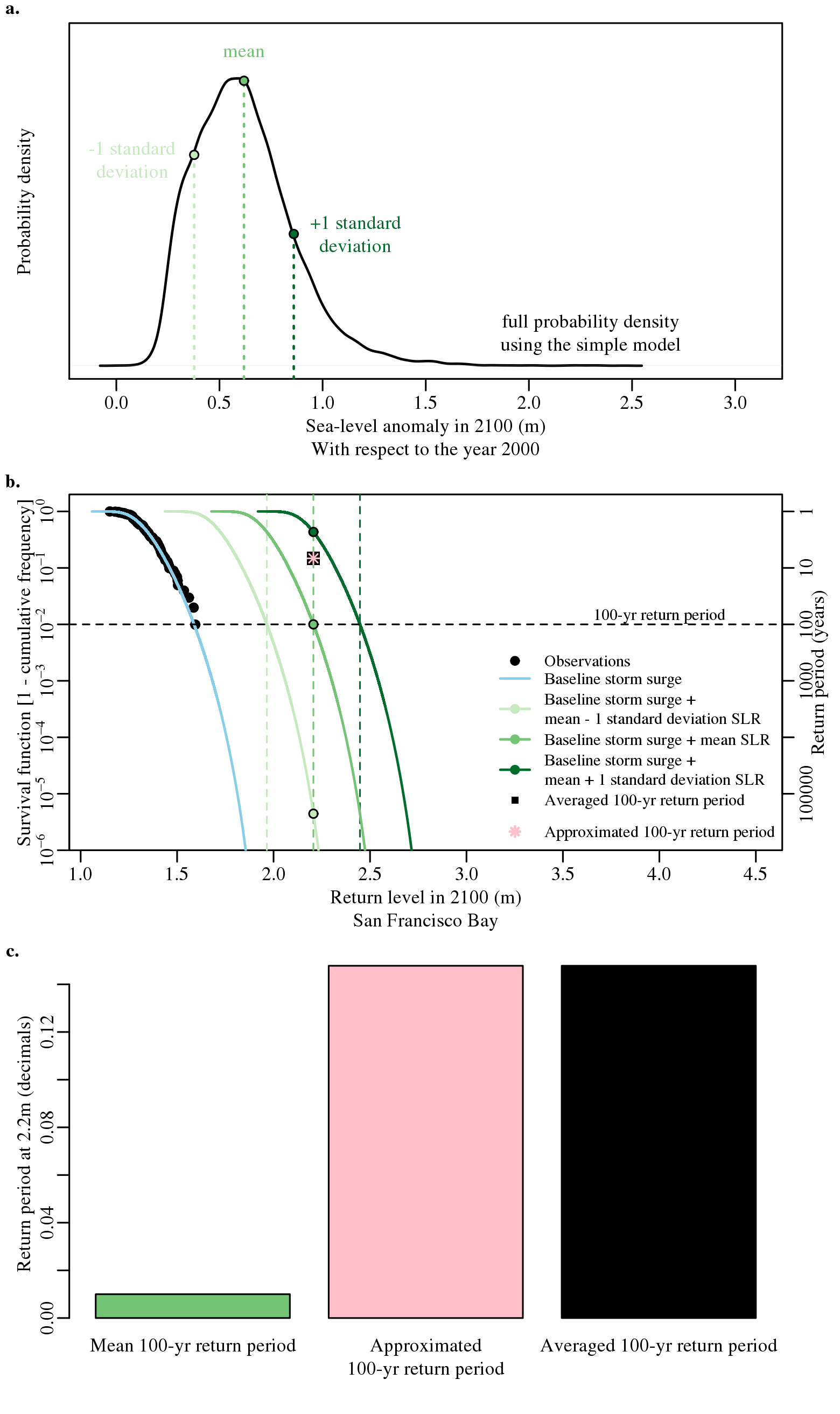

Supplement: S3 Fig — Panel a displays the probability density function of global mean sea-level rise in the year 2100 along with the mean ±1 standard deviation projections (green lines). In panel b, the baseline storm surge (light blue) is shifted by sea-level projections of 0.4 m (-1 standard deviation; light green), 0.6 m (mean; green), and 0.9 m (+1 standard deviation; dark green) to represent potential future flood height. The average of the three return periods at 2.2 m is represented as the black square. The pink star is produced when the return period below the 100,000-yr return period is approximated as zero and then averaged with the two other return periods at 2.2 m. Note that (panel b and c) the approximation method for the return period at 2.2 m produces the same return period value as the result from accounting for the return periods below 100,000 years and hence displays little to no flood risk over-or-underestimation. (TIFF) [file pone.0174666.s003.tiff]

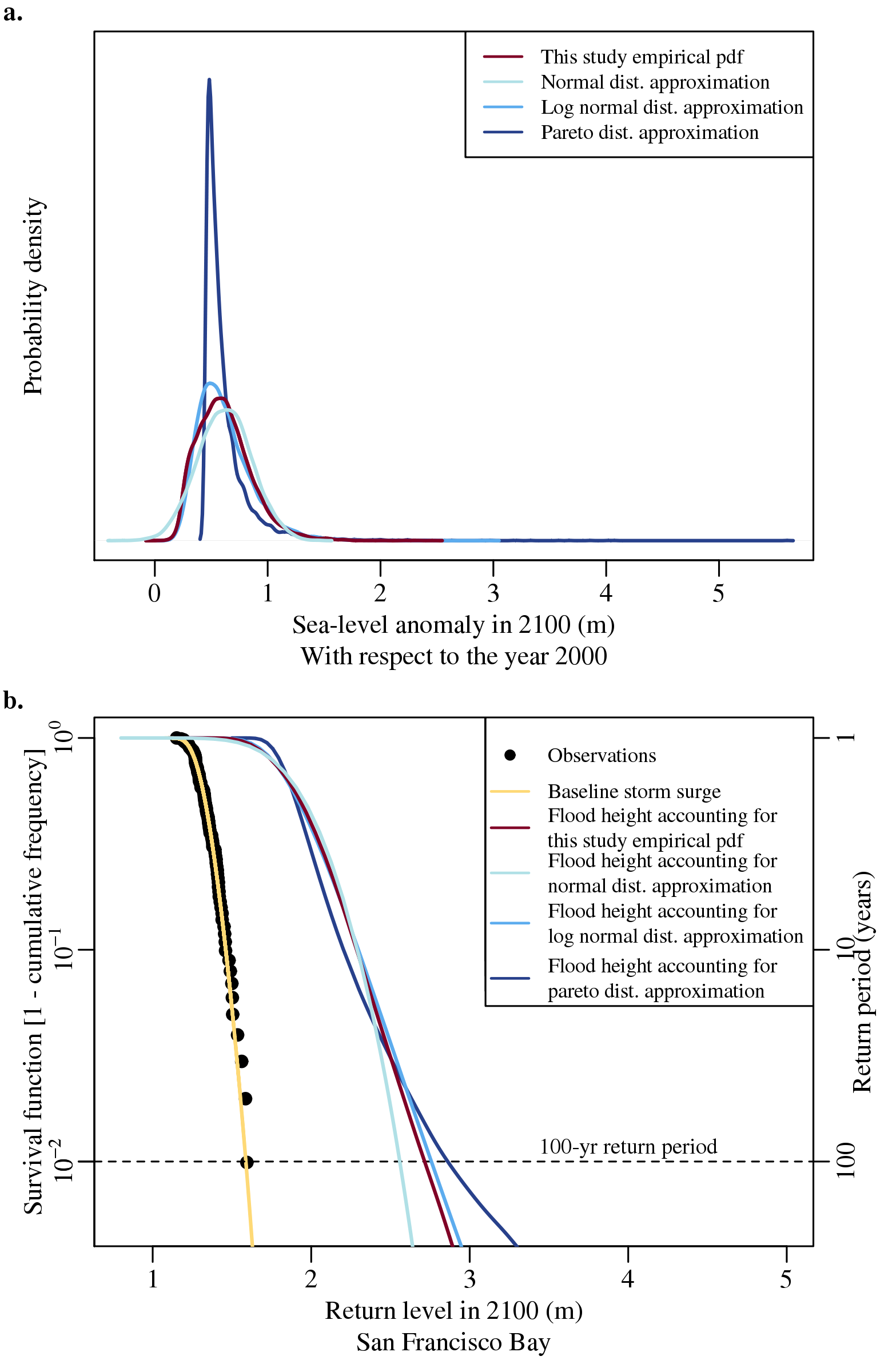

Supplement: S4 Fig — Panel a displays the probability density function (pdf) of our global mean sea-level rise in the year 2100 (dark red) along with the normal (light blue), log normal (blue), and Pareto (dark blue) distribution approximations of global mean sea-level rise in the year 2100. In panel b, the survival function accounting for each sea-level rise approximation (light to dark blue) is shown for comparison to the baseline storm surge (yellow) and the survival function accounting for our estimated empirical sea-level rise pdf (dark red). (TIFF) [file pone.0174666.s004.tiff]

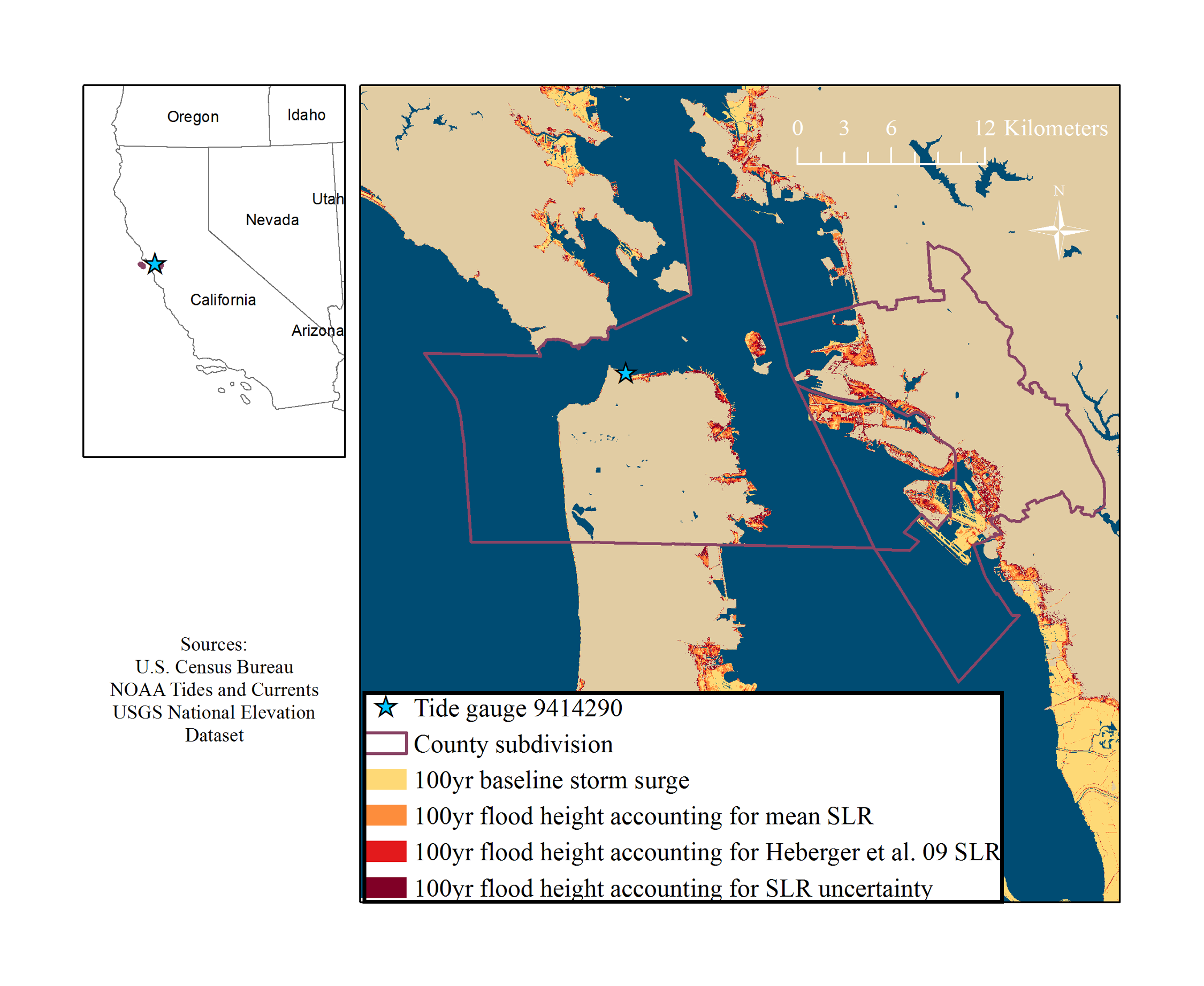

Supplement: S5 Fig — Each county subdivision (outlined in brown) is located within the analysis extent. In the analyzed area, the 100-yr flood risk area is displayed in yellow (baseline), orange (flood height accounting for the mean sea-level projection), red (flood height accounting for the Heberger et al. [7] sea-level projection; not accounting for land storage changes), and dark red (flood height accounting for sea-level rise uncertainty). The star represents the location of the tide gauge. (TIFF) [file pone.0174666.s005.tiff]

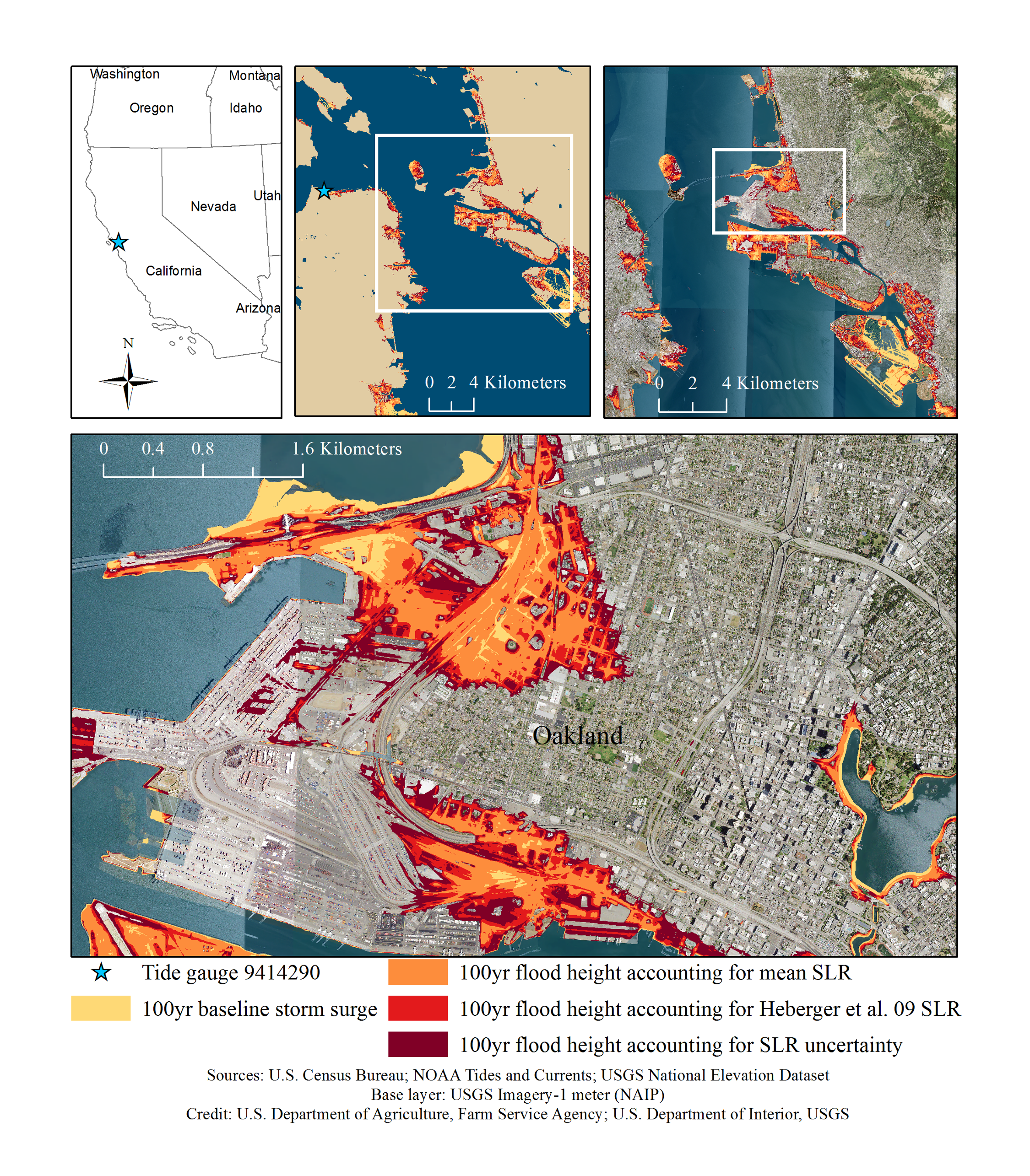

Supplement: S6 Fig — The maps display the baseline 100-yr flood risk area in yellow. In the year 2100, the potential 100-yr flood height includes the addition of sea-level projections. The future 100-yr flood risk area based on the mean sea-level projection is in orange, based on the Heberger et al. [7] (not accounting for land storage changes) sea-level projection in red, and based on accounting for sea-level rise uncertainty is in dark red. The location of the tide gauge is displayed as a star. (TIFF) [file pone.0174666.s006.tiff]

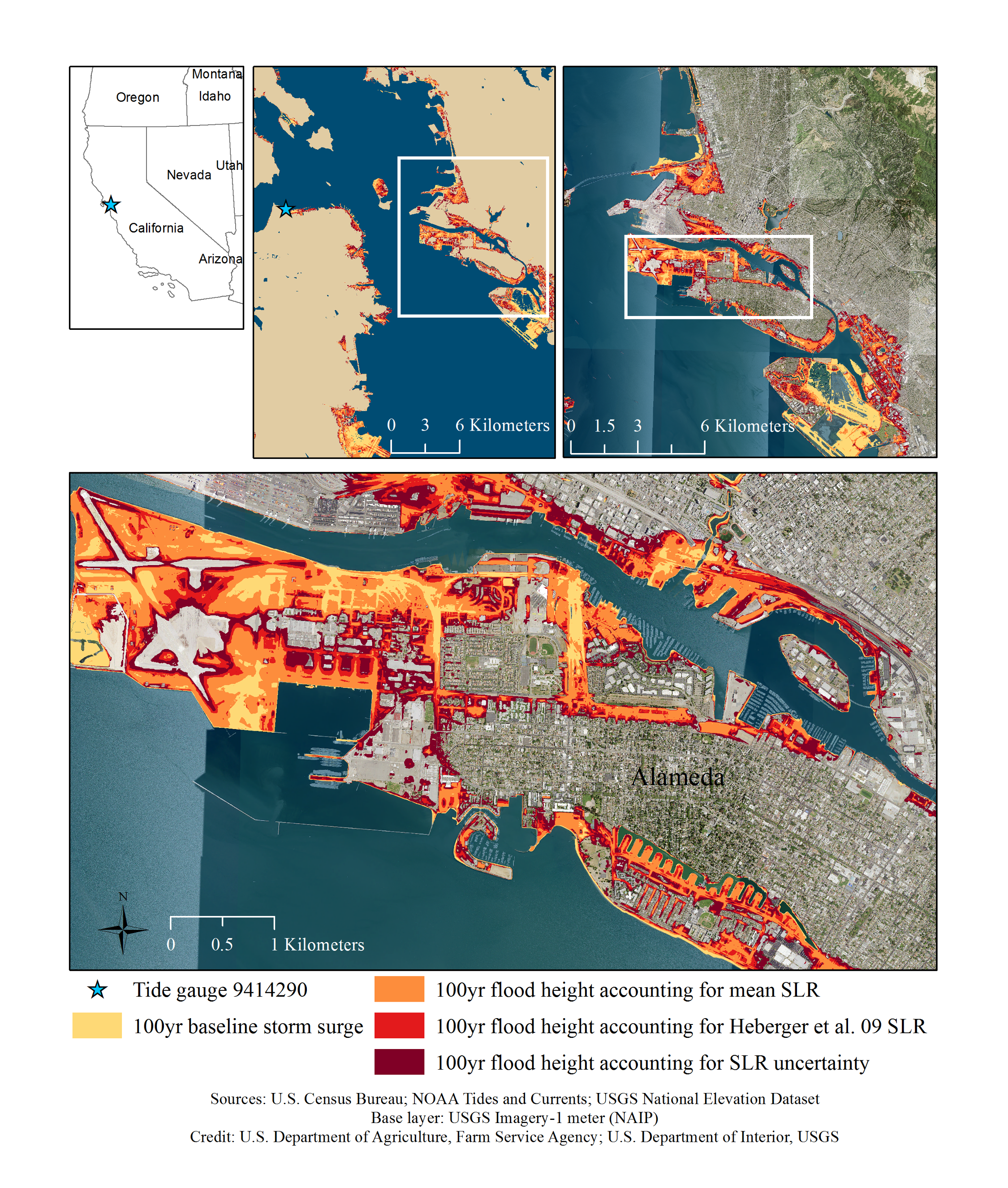

Supplement: S7 Fig — The maps display the baseline 100-yr flood risk area in yellow. In the year 2100, the potential 100-yr flood height includes the addition of sea-level projections based on the mean sea-level projection (orange), the Heberger et al. [7] (not accounting for land storage changes) sea-level projection (red), and accounting for sea-level rise uncertainty in dark red. The location of the tide gauge is displayed as a star. (TIFF) [file pone.0174666.s007.tiff]

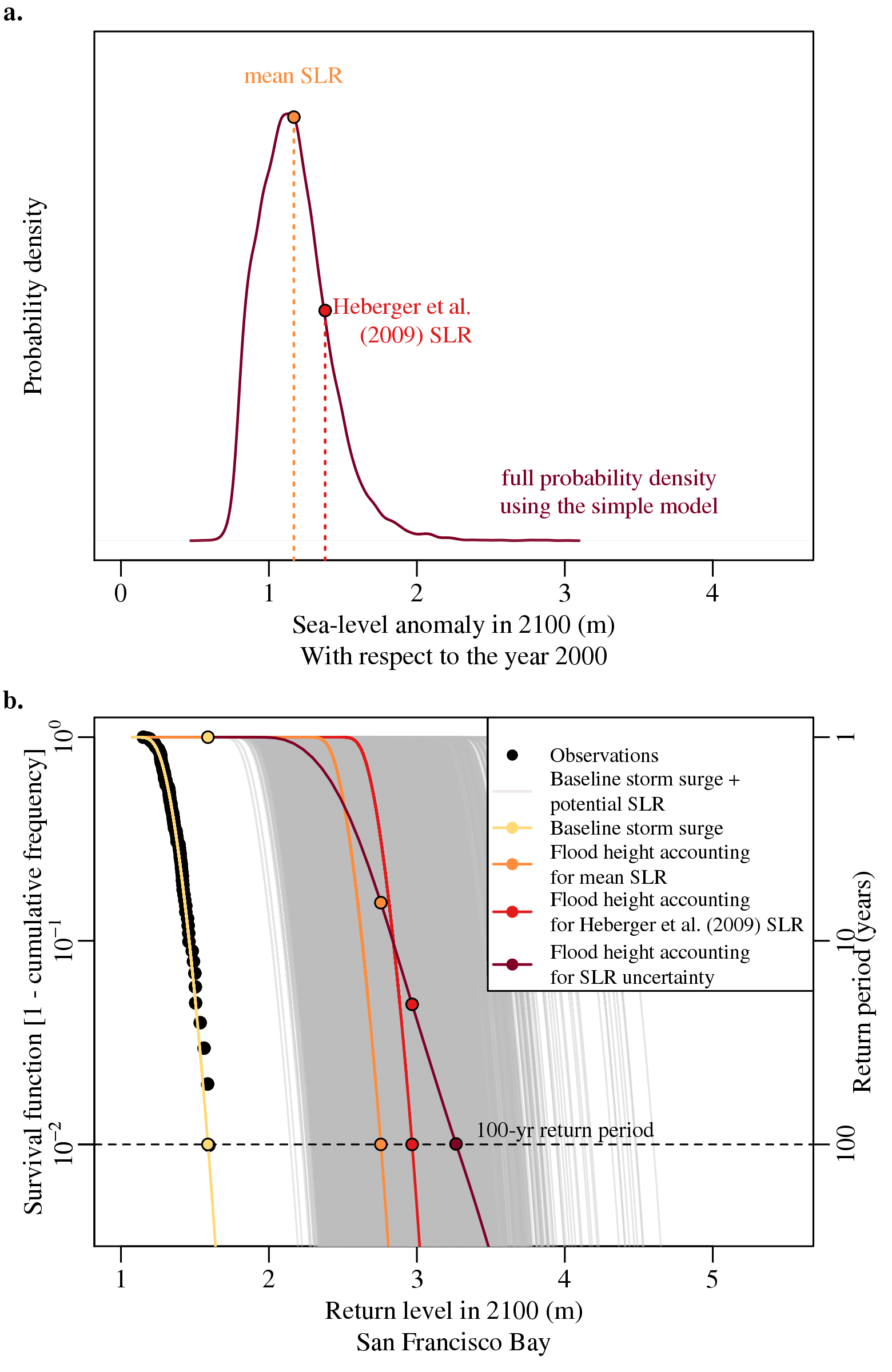

Supplement: S8 Fig — As in Fig 3, panel a is the probability distribution of global mean sea-level rise in 2100 and panel b is the flood survival functions for San Francisco Bay. However, in this figure we adapt our sea-level rise estimates in the year 2100 by adding roughly ∼0.55 m. This increase is roughly comparable to the increase used in Heberger et al. [7]. Ultimately, adapting sea-level estimates to this change does not change the main conclusions of this analysis. (TIFF) [file pone.0174666.s008.tiff]

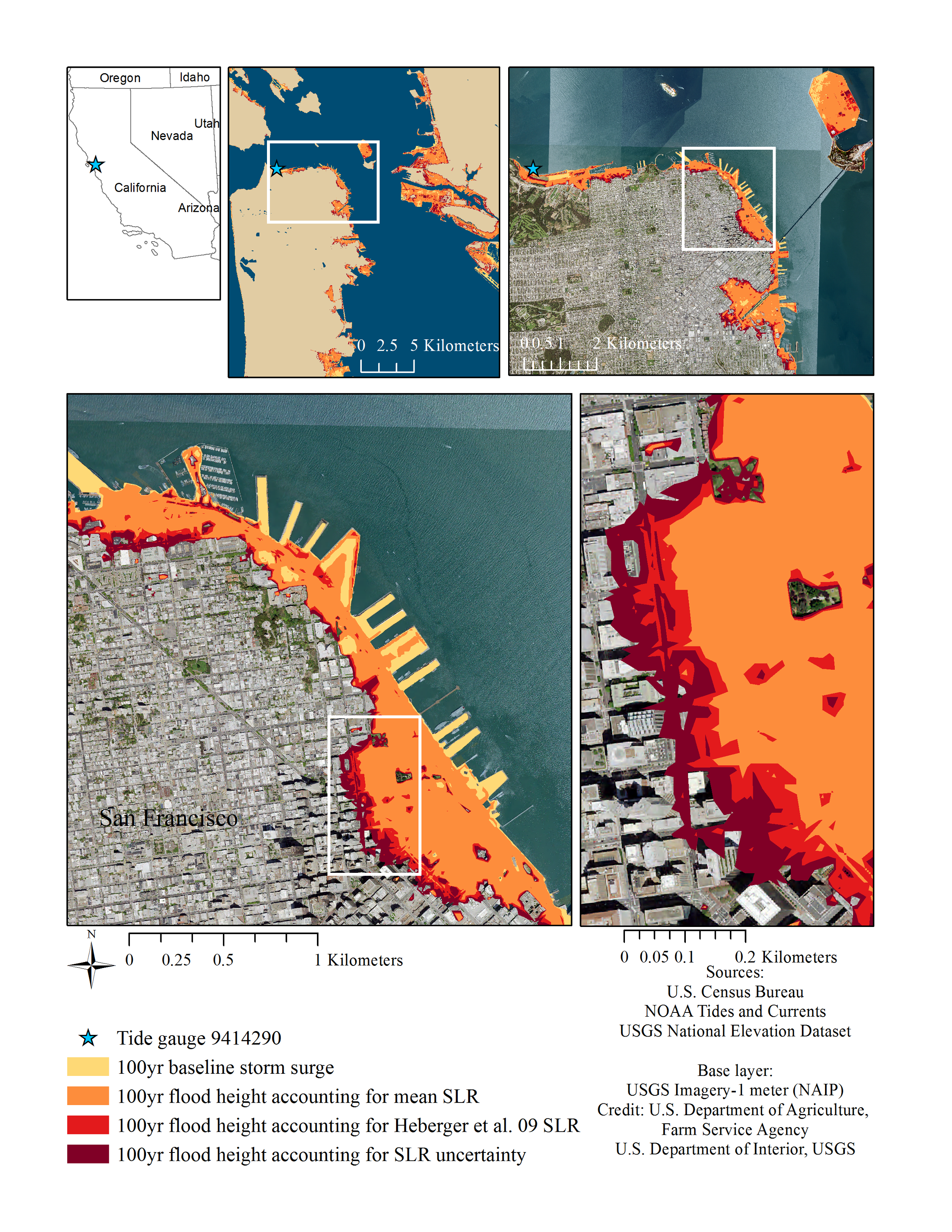

Supplement: S9 Fig — As in Fig 5, this figure shows a sequential zoom in of the baseline and future 100-yr flood risk areas in San Francisco. However, in this figure we account for changes in land water storage in the year 2100 by adding roughly 0.55 m to the sea-level estimates [7]. (TIFF) [file pone.0174666.s009.tiff]

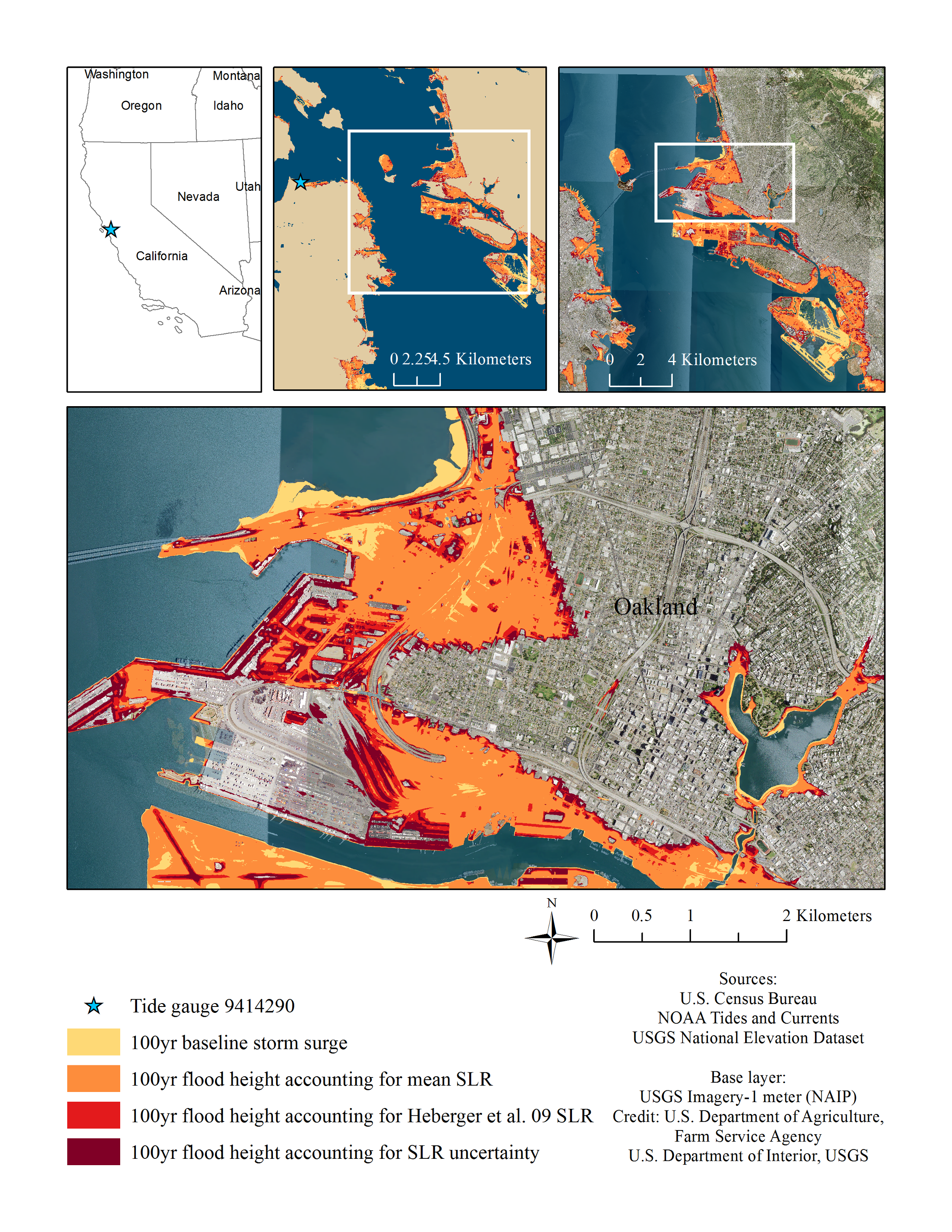

Supplement: S10 Fig — The maps of Oakland, CA display the baseline 100-yr flood risk area in yellow. In the year 2100, the potential 100-yr flood height includes the addition of sea-level projections. The future 100-yr flood risk area based on the mean sea-level projection is in orange, based on the Heberger et al. [7] sea-level projection in red, and based on accounting for sea-level rise uncertainty is in dark red. The location of the tide gauge is displayed as a star. However, in this figure we account for changes in land water storage in the year 2100 by adding roughly 0.55 m to the sea-level estimates [7]. (TIFF) [file pone.0174666.s010.tiff]

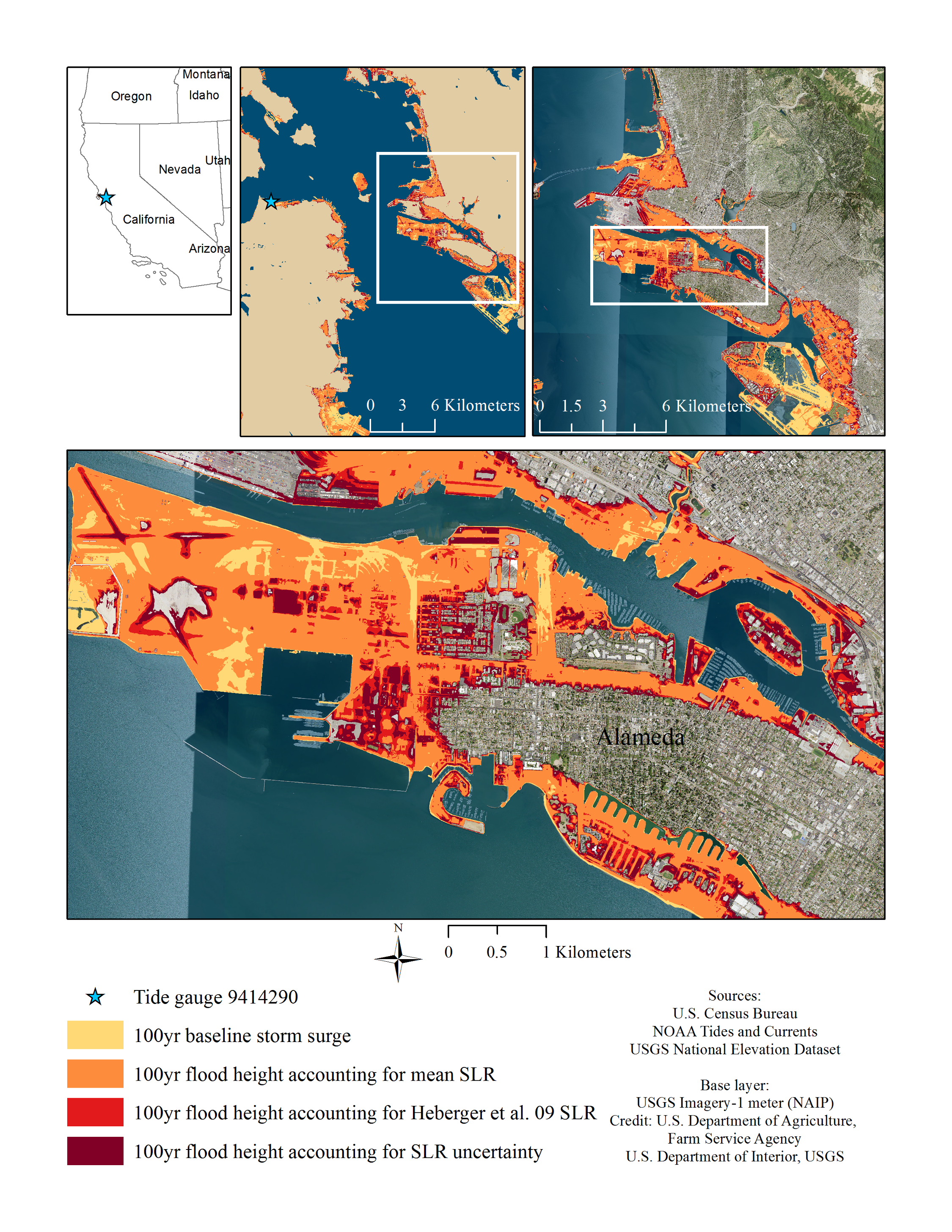

Supplement: S11 Fig — The maps of Alameda, CA display the baseline 100-yr flood risk area in yellow. In the year 2100, the potential 100-yr flood height includes the addition of sea-level projections based on the mean sea-level projection (orange), the Heberger et al. [7] sea-level projection (red), and accounting for sea-level rise uncertainty in dark red. The location of the tide gauge is displayed as a star. However, in this figure we account for changes in land water storage in the year 2100 by adding roughly 0.55 m to the sea-level estimates [7]. (TIFF) [file pone.0174666.s011.tiff]
